# Insights gained from a cultural adaptation of preschool promoting alternative thinking strategies©: the importance of teachers’ cultures as an implementation driver

**DOI:** 10.3389/fpsyg.2024.1425936

**Published:** 2024-08-07

**Authors:** Åsa Norman, Mina Sedem, Laura Ferrer-Wreder, Lilianne Eninger, Hanna Ginner Hau

**Affiliations:** ^1^Department of Clinical Neuroscience, Karolinska Institutet, Stockholm, Sweden; ^2^Department of Special Education, Stockholm University, Stockholm, Sweden; ^3^Department of Psychology, Stockholm University, Stockholm, Sweden

**Keywords:** child intervention, Sweden, social–emotional learning, implementation science, practice-based, pedagogy, education

## Abstract

**Introduction:**

Cultural adaptation of interventions is complex and yet vital to achieving the intended benefits of interventions with new populations. However, little is known regarding deliverers’ perceptions of cultural adaptation and when a cultural adaptation process can be considered complete. The purpose of this study was to explore aspects of cultural adaptation that need further attention in an intervention that had undergone an initial cultural adaptation.

**Methods:**

Four focus groups (FGs) were conducted with preschool teachers who had worked with a culturally adapted version of preschool Promoting Alternative Thinking Strategies (PATHS©) in Sweden for approximately 6 months. In total, 16 teachers from eight preschools were included, with 3–5 teachers in each group. All FGs were audio-recorded and transcribed verbatim. Thematic analysis with an inductive approach was applied to the transcribed data.

**Results:**

Three themes were identified where teachers described the need for further cultural adaptation for the intervention to align with personal and societal fundamental cultural values and be useful for their work as teachers in the Swedish preschool setting. The themes pertained to culturally adapting a manual-based intervention to a foundational, value-based approach, such as the practical application of core values and the steering documents of the Swedish preschool. Furthermore, the practical function of the culturally adapted intervention in the new cultural context revealed a further need to adjust materials and activities in interaction with the children. Finally, the prerequisites within the Swedish cultural setting, including resources and collaboration with parents as part of the work structure for preschool teachers in Sweden, needed further attention in relation to the intervention.

**Conclusion:**

The findings of this study highlight the importance of the deliverer in the cultural adaptation process in addition to adaptations that focus on end users (children in the case of preschool PATHS). Furthermore, the study indicates a need for a more open-ended view of the cultural adaptation process for interventions than perhaps previously described in models of cultural adaptation of interventions.

## 1 Introduction

An intervention’s cultural relevance is crucial to realizing beneficial changes. It also connects with other important aspects of implementation, such as participant and facilitator acceptability and engagement (Barrera et al., 2017). This is the case when an intervention is developed within a single multi-cultural nation as well as when interventions are imported for use across nations, which also may be multi-cultural. In these situations, there are unique opportunities to understand patterns of health among socially and culturally important groups of individuals, with insights to be gained that can help to achieve wide-scale impact and benefits (Kumpfer et al., 2017; Soto et al., 2018).

Intervention cultural adaptation refers to systematic modifications of interventions to align intervention language, content, and context with participants’ culturally rooted values and patterns (Bernal et al., 2009). The field of interventional cultural adaptation has grown substantially during the past decades, informing the wider field of intervention science regarding how to carry out systematic adaptations with scientific rigor. An adaptation can entail (a) skipping or removing part of an intervention, (b) modifying an existing part of an intervention, or (c) adding new intervention activities or content that leaves the rest of the intervention largely unchanged (Soto et al., 2018). Finally, adaptations can be of a spontaneous or planned character (Barrera et al., 2017). The field of implementation science largely considers and measures intervention adaptation. Adaptation has been debated in relation to both the surface and the deep structure of interventions. Intervention surface structure is, for example, the appearance of the intervention, such as language and layout of material. Whereas intervention deep structure or intervention core components transform an intervention’s theory of change into an action that can benefit people (Resnicow et al., 2000; Ferrer-Wreder et al., 2012). Here, a meta-analysis of culturally adapted parenting programs indicated that adaptation of intervention deep structure rendered the largest beneficial effect sizes (van Mourik et al., 2017).

Within the field of intervention and cultural adaptation, several frameworks have been posited as aids to facilitate the process of cultural adaptation of interventions (Domenech Rodríguez and Bernal, 2012). Two commonly used frameworks were proposed by Bernal et al. (1995) and Castro et al. (2004). The framework by Bernal et al. (1995) emphasizes the consideration of the following aspects: language, persons, metaphors, content, concepts, goals, methods, and context. According to the framework, *language* should be considered beyond mere translations in favor of in-depth cultural knowledge and norms. The consideration of *persons* refers to capturing the cultural similarities and differences between the participant and deliverer, whereas *metaphors* refer to concepts and symbols relevant to the cultural context. The aspect of *content* refers to cultural knowledge and the aspect of *concept* highlights that theoretical concepts used in the intervention must align with the cultural context. Furthermore, participants and deliverers agree on the *goals* of the intervention, the *method* used as intervention needs to suit the cultural context, and finally, *contextual aspects* pertaining to the personal situation of the participant but also the surrounding economic and political context need to be considered. The framework put forward by Castro et al. (2004) emphasizes three aspects of guiding adaptation strategies to achieve participant acceptability. The first aspect refers to *cognitive information processing* characteristics of the participants, such as their language and age. The second aspect refers to the *affective motivational* characteristics of the participants, which may require substantial modification of intervention activities to align with culturally related values in the new context. The third aspect, *environmental* characteristics, refers to aspects of the community context. In addition to the framework by Bernal et al. and Castro et al., the Planned Intervention Adaptation (PIA) framework is another approach to the cultural adaptation of interventions (Ferrer-Wreder et al., 2012). Concisely put, PIA consists of an a priori approach in which coalition-building efforts are encouraged and the results of formative research studies guide a reflection on intervention fit to the new context. These efforts and study results act as a basis for making planned adaptations to the intervention, which is then later tested in a controlled intervention trial. Other features of PIA are that it could be used with various types of interventions and that it is cross-nationally focused. Furthermore, the PIA framework has a view of culture as complex, far-reaching, and intervention-specific and identifies that important aspects of culture could enter into the intervention itself and thereby benefit the intervention by adapting both its deep and/or surface structure (Resnicow et al., 2000). The PIA framework also identifies that program stakeholders, including intervention facilitators, program developers, and end users, have a vital, participatory research role in helping researchers determine what should be adapted or retained in the intervention cultural adaptation process (Bernal et al., 2009).

Thus, important progress has been made within the field of interventional cultural adaptation. There are, for instance, several adaptation frameworks available and better documentation of the cultural adaptation process itself when culturally adapted interventions are tested. Yet, little is known about the extent of optimal and practical adaptation and when the adaptation process can be considered complete. Most frameworks and studies to date focus on the importance of adapting interventions to the cultural patterns and values of the intended end users who will ultimately benefit from the intervention. Yet, the cultural adaptation of interventions in relation to the cultural understanding and values of intervention deliverers has received comparatively less attention. Deliverers and those who support implementation play key roles in making an intervention happen by delivering the intended benefits of an intervention and making interventions available to end users in practice. In the field of implementation science in general, without the additional consideration of what is needed for cultural adaptation/relevance, it is well known that intervention deliverers need specific consideration. Reasons for this specific focus is that deliverers often struggle with demands related to high-fidelity and dosage-intensive interventions, based on several factors related to the intervention itself, the organization, resources, and personal factors (Damschroder et al., 2009; Aarons et al., 2012).

Promoting Alternative Thinking Strategies (PATHS©) is an example of an intervention that is widely used and has been implemented and tested within several cultural contexts. The PATHS intervention is a U.S.-developed school-based, teacher-implemented universal prevention program aimed at supporting child and adolescent social–emotional learning (SEL) (Greenberg and Kusche, 1998; CPPRG, 2010). In light of the generally positive evidence base for PATHS shown in the United States (e.g., Domitrovich et al., 2007; Bierman et al., 2008), PATHS (preschool and elementary school editions) have been culturally adapted and tested in several countries. In Hong Kong, a cultural adaptation of the elementary school edition was performed without guidance from a stated *a priori* cultural adaptation framework and resulted in several adaptations of primarily surface structure, such as changes to material within the culturally adapted lessons connected to emotions (Kam et al., 2011). The adaptation of preschool PATHS to a Pakistani context was guided by the framework proposed by Castro et al. (2004) (Barrera and Castro, 2006). Here, the nature of the adaptations was also described as mainly of a surface structure character, including changes in language, material presentation, intensity of delivery, and the use of culturally compatible concepts (Inam et al., 2015). In Sweden, the preschool edition of PATHS was culturally adapted using the PIA framework (Ferrer-Wreder et al., 2021), which also rendered adaptations that were mainly of a surface structure nature, such as translations, terminology related to some activities, material, and training procedures (Ferrer-Wreder et al., 2021). As PATHS is widely tested across several contexts and has undergone several cultural adaptations, it serves as a good example of an intervention from which further knowledge on the nature of cultural adaptation processes and the role of the deliverer of the intervention in such a process can be explored.

In summary, the cultural adaptation of interventions is complex and multi-faceted and vital to achieving deliverer and participant engagement and effectiveness, and thereby achieving the intended benefits of interventions widely and with new populations. As noted, several frameworks have been developed to guide such adaptation processes. However, less attention has typically been directed to deliverers and to the consideration of when a cultural adaptation process can be considered completed or if this process is better viewed as open-ended and not just mainly a pre-intervention trial activity.

This study aimed to explore the aspects of cultural adaptation that need further attention in an intervention that has already undergone an initial cultural adaptation. We investigated the perceptions of teachers who delivered the preschool edition of PATHS, which had been culturally adapted for use in an urban Swedish preschool context.

## 2 Materials and methods

### 2.1 Study design

This study used a qualitative descriptive design, which permits studying perceptions and patterns in-depth (Patton, 2015).

### 2.2 The PATHS intervention

Promoting Alternative Thinking Strategies was originally developed in the United States and is a widely implemented SEL curriculum/intervention (Greenberg and Kusche, 1998; CPPRG, 2010; Calhoun et al., 2020). PATHS is usually teacher-implemented universally in classrooms and other school settings (e.g., playgrounds). In terms of content and organization, PATHS’ progression of lessons (33 in preschool PATHS) and activities focuses on fostering children’s emotional knowledge and awareness, as well as supporting the development of skills concerning relationships, problem-solving, and self-regulation (Domitrovich et al., 2007). Preschool PATHS lessons are designed to be implemented regularly throughout an academic school year. The lessons are interactive with the use of puppets, role plays, story books, feeling face cards, and giving and receiving compliments. Lessons also include the use of self-calming techniques that aid children in gaining a deeper understanding of their own emotional life and serve as tools to direct their behavior in a self-directed way (Domitrovich et al., 2007). PATHS is designed to assist teachers in strengthening their classroom climate in ways that are consistent with an intentional focus on promoting children’s social–emotional competence (Domitrovich et al., 2007). In addition to the curriculum, there is a manualized supporter model that entails semi-structured visits with teachers at the preschool from coaches/supporters.

In Sweden, PATHS was tested in a cluster-RCT during 2014–2016 in 26 preschools, including 285 children aged 4–5 years (Eninger et al., 2021). Prior to the RCT, a cultural adaptation process using the PIA framework (Ferrer-Wreder et al., 2012) was undertaken. Concisely summarized, the PIA process included coalition-building efforts that took place in the selection of the intervention to be used, the results of the FGs with an expert group of teachers (not the same group of teachers as in the present study), and two small scale quantitative cross-sectional studies (Ferrer-Wreder et al., 2021). Consistent with other prior adaptations of PATHS in Hong Kong and Pakistan (Kam et al., 2011; Inam et al., 2015), for the Swedish intervention trial, the cultural adaptations were mainly of a surface structure nature (Ferrer-Wreder et al., 2021). The adaptations included translation from English into Swedish of all intervention curriculum materials, reframing terminology around the use of compliments as part of the intervention as reflected in the training guidance with teachers, and issues around the portrayal of gender in intervention materials. The adaptations also included changes to the intervention training sessions so that teachers had a clearer message about the prescriptive elements of PATHS and the degree of openness of these elements to modification based on teachers’ needs and pedagogical approach.

The RCT following the cultural adaptation in Sweden showed primarily beneficial outcomes and also involved unique benefits for certain groups of children. More specifically, girls and children attending preschool in neighborhoods in which residents had lower and higher incomes had greater benefits from participating in PATHS (Eninger et al., 2021; Kapetanovic et al., 2022). Implementation data showed the average teacher-reported lesson coverage as 14.8 lessons, which represents an average of 45% of the entire curriculum reported to be covered by teachers. However, the variation was large, and there was 50% missing data on the report of teacher lesson coverage. Supporter and independent rater assessments of teachers’ fidelity to PATHS were on average 3.6 on a five-point scale (between *3 = neutral* and *4 = does pretty well*) (Eninger et al., 2021).

### 2.3 Setting of the present study

The cultural adaptation of PATHS using the PIA framework was conducted in the context of Swedish early childhood education and care (ECEC) in a large urban city. In Sweden, the ECEC context this study was conducted in is referred to as preschool. Swedish preschool is a part of the national school system and is regulated by the Education Act and the National Preschool Curriculum (Swedish National Agency for Education, 2018). Preschool is publicly subsidized, and all children aged 1–5 are legally entitled to full-day provisions. Even if preschool is not mandatory, attendance is typically high. For example, according to the Swedish National Agency for Education (2022), 86% of all children in the target population attend. The quality of ECEC in Sweden is ranked highly globally. In the report of UNICEF (2008), no country but Sweden had a full benchmark for ECEC. As in the other Nordic countries, this access to ECEC is part of what forms the welfare model, often referred to as the Nordic model or the social democratic model (Ferrer-Wreder et al., 2020). As a reflection of democratic values such as solidarity, civil rights, equity, and equality, children are considered to be a responsibility not only of their families but also of society. Consequently, ECEC, just like school education, is regarded as a matter of societal importance (Broström et al., 2018). The qualifications for staff in ECEC are either a university degree (3.5 years), which more than half of staff have or 2–3 years of upper secondary/senior high school training (Samuelsson and Sheridan, 2004). According to the Swedish National Agency for Education (2022), 96% of employees in Swedish preschool are women.

### 2.4 Data collection and participants

Focus group methodology was deemed appropriate as a method for data collection. In FGs, data are generated through the interaction among participants in the group, which makes it suitable for studying perceptions and experiences (Krueger, 1994; Kitzinger, 1995).

A convenience sample of teachers who were trained in the Swedish cultural adaptation of preschool PATHS and had been using the intervention for some time was included in this study. Teachers in all 14 preschools who participated in the PATHS intervention trial were invited to participate in the FGs. For this study, the teachers needed to invite to participate in the FGs. They had some experience from working with the Swedish cultural adaptation of PATHS to be able to express their experience of it. In total, 16 preschool teachers from eight different preschools chose to participate, whereas others denied participation due to a lack of time. All participants were women with a variation in years of experience working in the preschool context. The preschool teachers had all worked with a culturally adapted version of preschool PATHS for approximately 6 months (Ferrer-Wreder et al., 2021). Four FGs were conducted during the spring of 2016, when the intervention trial testing a culturally adapted preschool edition of PATHS in Sweden was ongoing. Each FG included three to five preschool teachers from the eight different preschools and were moderated by female researchers or research assistants, of whom none are authors of this study. The moderators were part of the research team that conducted the Swedish preschool PATHS and had met with some of the teachers prior to the FGs. FGs lasted for 54–76 min with a mean of 60 min. The FG discussions were guided by one moderator and one assistant using a semi-structured interview guide with open-ended questions to facilitate interaction and discussion among participants. Examples of questions in the interview guide were: How did you perceive the work with PATHS overall? What were the benefits and challenges of working with PATHS? What possibility did you have to integrate the basic ideas of PATHS with your daily work routine with the children? What resources are needed to implement PATHS in preschools? The interview guide is available as Supplementary material 1. All FGs were audio-recorded. Ethical approval for this study was approved by the Stockholm Regional Ethics Review Board (Dnr. 2012/1714-31/5). Written informed consent to participate in this study was provided by participants.

### 2.5 Data analysis

The digital audio from the four FGs was transcribed verbatim. The transcripts were imported into Nvivo12 and Open Code for qualitative data analysis by two female coders (MS and ÅN). Both coders are experienced researchers in qualitative methods and trained in psychology and behavioral sciences. To decrease bias, the researchers/coders were not moderators of the FGs and did not transcribe the digital audio from the FGs. The data analysis was guided by principles from thematic analysis (Braun and Clarke, 2006), which can be carried out in different ways, either deductively by theory, inductively by empirical data or a combination of inductive and deductive approaches (Hayes, 2000). In the current study, the transcripts were examined using an inductive approach by focusing on identifying themes from empirical data guided by the study’s aim. No conceptual categories for coding were established *a priori* (Hayes, 2000). Initially, the coders read the transcripts thoroughly to familiarize themselves with the data. Each coder separately used an inductive bottom-up approach and conducted the initial coding. They read each FG transcript and assigned codes in a descriptive word or phrase to label significant ideas in the participants’ comments. Using the software (Nvivo12 and Open Code) to manage the data, the coders could organize the data in groups, link relevant codes and quotes, create a code list and cases by coding sections of a file, use different tools such as text search Query coding, compare and contrast text passages, look for similarities and differences, and also write memos to generate interpretations. The coders discussed and compared codes throughout the iterative coding process. After coding the transcripts, each coder compared the codes and combined the codes to make distinct themes in an iterative process. In the process, the coders discussed the emerging theme structure, and decisions were made about preliminary main and sub-themes. Then, each coder tested the preliminary themes by recoding all data with the aim of confirming that themes captured the essence of the FG interviews. As a result, three themes with two sub-themes each were finally agreed upon. In the final step, results were written and reviewed to ensure that each theme was coherent and that all themes provided a rich and significant picture of the participants’ views. Illustrative citations were given for each theme. Participants are labeled teacher (T) with an assigned participant number when quoted in the text, with the number of the FG added at the end of the quote. In order to check the study for quality and content, the COREQ checklist for interviews and FGs was used (Tong et al., 2007) (Supplementary material 2).

## 3 Findings

**Figure 1:**
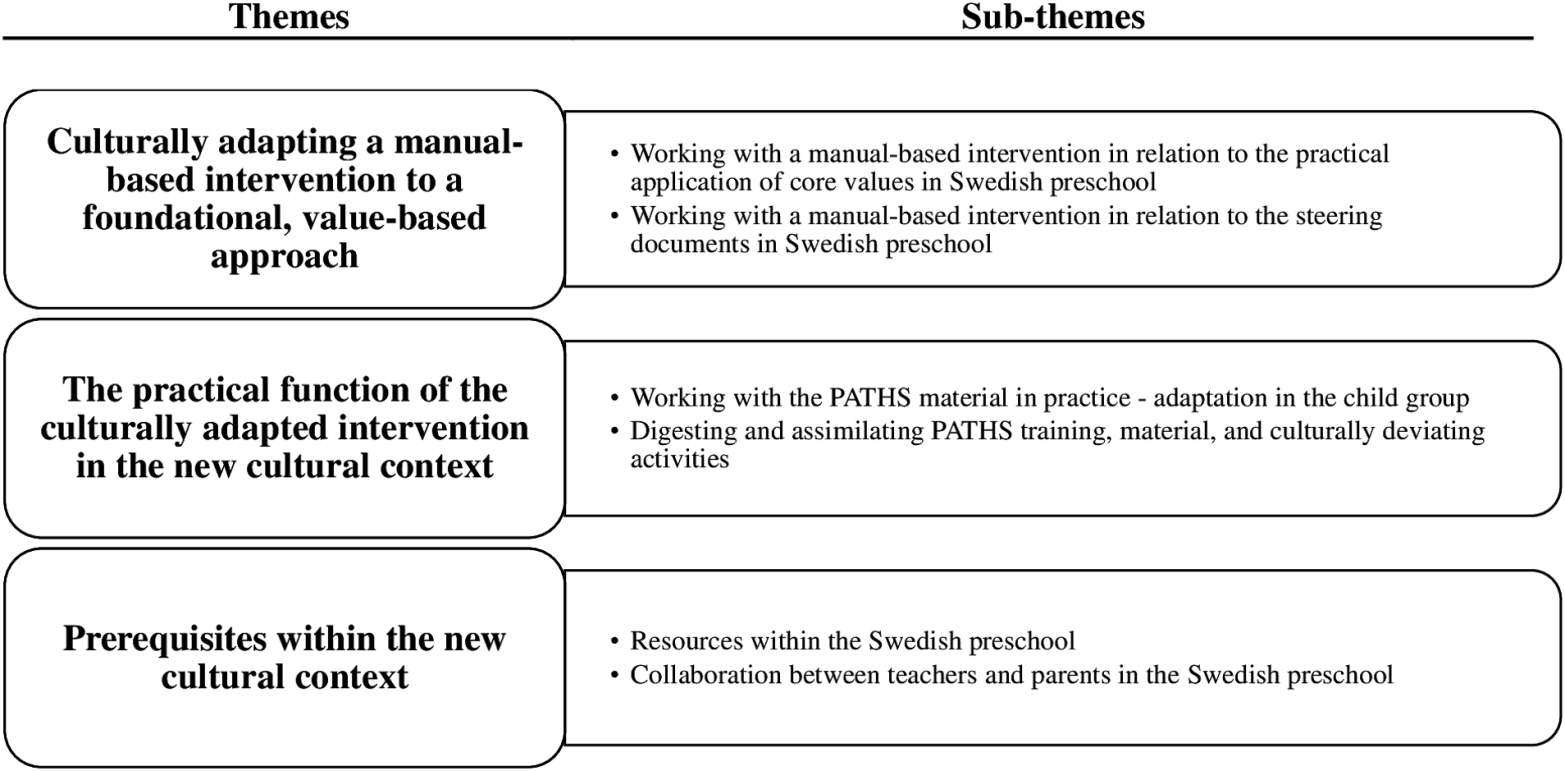
Themes and sub-themes as identified in the thematic analysis.

The participants’ perceptions of aspects of the intervention that needed further cultural adaptation were identified as three themes presented in the text below and in Figure 1.

### 3.1 Culturally adapting a manual-based intervention to a foundational, value-based approach

This theme encompassed the relationship between the culturally adapted preschool PATHS intervention and the underlying basic values of Swedish preschool as perceived by participants. The teachers discussed how PATHS needed further cultural adaptation in relation to the central focus on children’s needs that permeates the work in preschool, both in terms of practical pedagogical work and in terms of the steering documents for preschool.

#### 3.1.1 Working with a manual-based intervention in relation to the practical application of core values in Swedish preschool

The teachers discussed the fundamental view of children within the Swedish preschool system, how this view is expressed in both pedagogy and the structure of the preschool and how they consistently apply this central value in the practical everyday work at preschool. This fundamental view emphasizes that all children should have the possibility to participate in preschool activities regardless of, e.g., disability or social needs, and how this value could have been strengthened in the possible further adaptation in the version of PATHS that they worked with.

The teachers discussed the fundamental view that children’s needs are at the heart of pedagogy and that great pedagogical flexibility is required as preschool activities are to be adapted to all children. Here, the teachers expressed how they prioritized children’s needs most and how they work to be responsive to children rather than seeking to take a teaching role, which they perceived that the PATHS manual emphasized more. Some teachers felt that PATHS’ fundamental pedagogy ran counter to the Swedish fundamental view of children’s participation in activities and interaction between the teachers and the children rather than the teachers taking a governing, steering role.

“T4: So in my view, it feels outdated. I mean, they talk about lessons. […] There is a teacher, who is teaching. But that is not how I am taught [to work as a preschool teacher]. What I learned was that you are a co-investigator. That means that we create things together. I mean, it is less about copying, that children should copy adult ways of thinking. It is more about creating new alternative ways of thinking that come from the children […].”

T1: We have a different educational philosophy [than the American one]. I mean we don’t have a school like it seems like they do [in the US].

T4: But we have a different view of children. […]

T3: In particular, the rules for circle time, [I was] a bit horrified too, a bit of this old school, to raise your hand […], circle time for me is a moment where we are together and do something together… FG 2.

The teachers also discussed that individual children and child groups may have very different needs based on disabilities, social vulnerability, or migration status, which further requires flexibility in pedagogy, e.g., regarding languages for newly arrived children or strategies for children with externalizing behavior. The teachers described that it could be difficult to use PATHS manual-based activities to cover the broad flexibility that children’s varying needs require.

“T2: We have had a problem there. Because we have one hundred percent children [whose] parents have come [immigrated] to Sweden. And the language, we also have children who are entirely new arrivals and do not know a word of Swedish. We have to work a lot with adapting the language. So, we really need to go through and look at the material and see, ‘But that is not possible! That I have to simplify’ or … […] I try to include the hand puppets much more, in what we are working on right now so that the children… well, they respond so well to the dolls. So, in that sense, we have really had to think about how to use the material”—FG 3.

Based on the central value that the needs of the child group should govern the preschool work, the teachers perceived that they have great individual responsibility for planning the content of the activities to suit the needs of the specific child group. The teachers found that working with a structured manual was somewhat unfamiliar, inconvenient, and, to some extent, degrading their competence. They reflected on how the Swedish preschool is largely based on the fact that the teachers have the competence to determine the needs of the child group and adapt the activities accordingly. Thus, the teachers had difficulty in identifying how the manual was to be implemented in its entirety.

“T4: We [teachers] don’t like someone putting a script in our throats, and that we are just supposed to do as someone tells us to do it. We do what we want to do, and we will continue to do it. […] I am damn proud of how I have approached PATHS. I have taken about 10–15 percent, which I embrace and use every day. And that is how it should be. That is how I want thing works in Sweden at least-no one can come and say to me, ‘Now you will do this all semester, straight off’”—FG 2.

#### 3.1.2 Working with a manual-based intervention in relation to the steering documents in Swedish preschool

The teachers discussed how PATHS fit with the National Preschool Curriculum, which regulates the overall mission of preschools, core values that should pervade the work in preschools, and goals and guidelines for the work. The teachers’ perceptions of whether PATHS fit the National Preschool Curriculum well differed. On the one hand, teachers described how PATHS’ content aligned with parts of the preschool curriculum and thus with work that the teachers were required to do with the children related to emotions, empathy, and friendly behavior. On the other hand, parts of PATHS were perceived to contrast with the focus on democratic processes in the Swedish curriculum. The curriculum emphasizes children’s influence and participation in decisions in accordance with a democratic agenda. The teachers experienced that activities such as the way child groups were governed or rules were decided upon according to PATHS contrasted the democratic process described in the National Curriculum.

*“T2: [The] biggest question [is] how we should intertwine PATHS with the National Preschool Curriculum. How should we conduct PATHS in a more democratic way? For example, with the rules, it is almost as if you are told ‘Do this, do that. Sit like this. You are supposed to sit and do this and that’. It is a bit authoritarian”—FG 1.*

### 3.2 The practical function of the culturally adapted intervention in the new cultural context

The theme encompassed the teachers’ experience of the practical work and their assimilation of the culturally adapted PATHS with regard to how the activities, material, and training needed further adjustments to allow for smooth practice in the Swedish preschool context.

#### 3.2.1 Working with the PATHS material in practice—adaptation in the child group

The teachers described the material as useful in general but expressed a consistent need to adjust PATHS activities to fit the Swedish preschool context. Adjustments were based not only on the children’s needs but also on the teachers’ own preferences, as well as already established work procedures regarding social–emotional competence that teachers used in their day-to-day work.

The teachers described how they adapted the PATHS material to the specific child group’s needs. Here, teachers also discussed whether the maturity of 5-year-old in Sweden differed from that of the United States, as they identified that the children got bored by PATHS activities intended for their age. Overall, adaptations to children’s needs were expressed through their reactions to the material and activities, e.g., children losing patience and interest when having to wait a long time for their turn in an activity.

“T2: Well, my whole group becomes one big adaptation (laughs). I have tried to have the star of the week, but it is not working well… the child almost loses interest with these days going by, and the others wait and wait until it is their turn”—FG 4.

The teachers also perceived a need to perform the activities in an adapted manner that they felt comfortable with and adapted to work procedures that they already used in their work with social–emotional competence. The teachers adapted PATHS to existing work procedures that were either based on a method or the teachers’ ideas. It was essential to the teachers that the PATHS material aligned with the teacher as a person, in line with the teacher being used to designing activities themselves to suit the children’s needs. The teachers expressed that they had to put quite some work into making the material “their own” and actively chose to make adaptations based on their preferences and included external material as needed.

“T4: Well, it is about how one is as a person, how one functions.

T5: You choose where you start from and don’t have to be so punctual but take whatever is adjusted to your child group.

T4: Well, you take what you want, kind of, and then you adapt it to your own knowledge.

T2: And it feels to me like, ‘This is what you should do’. Well, I have really made it my own material, and I do what I believe […] this feels right for me, and I think it gets applied to my group of children”—FG 2

#### 3.2.2 Digesting and assimilating PATHS training, material, and culturally deviating activities

The teachers perceived that linguistic and culturally profound differences reflected by certain PATHS activities needed further cultural adaptation in order to facilitate their use of these parts of the intervention.

Regarding language, the teachers described that having the PATHS training partly conducted in English was a challenge and that having training in Swedish would have facilitated their understanding of PTAHS. The training sessions in English forced them to focus more on understanding the information than on understanding the core of the PATHS intervention and how it could be applied to their everyday work. As for the PATHS material, the translations were partly perceived as flawed, with the result that some teachers found it difficult to adhere to the activities as described in the manual.

“T3: I thought it was a quite bad translation in Swedish. I must say, it was not a very good translation. It was a bit difficult to understand everything.

T2: Yes, there is a bit of wrong word order or wrong words in some places. It feels like words you don’t usually use.

T1: I haven’t thought about it too much. I have taken bits and pieces of what we thought was necessary out of it and not thought about that. It is a matter of making it your own, so to speak. So, I suppose that is the challenge really.

T2: Exactly, you cannot read verbatim when you are doing it in front of the kids. It is as someone said, you have to read and try to tell it in your own words sometimes, to make it more alive”—FG 4.

Furthermore, some specific activities were found more difficult to adhere to due to cultural differences and needed further cultural adaptations. Examples of such activities were books and songs with a firm base in the North American context that would have needed a stronger Swedish contextual connection. One activity that was experienced as specifically difficult due to cultural differences was the use of compliments. On the one hand, teachers attempted to use the compliment activities to various extents, and compliments were appreciated by both teachers, children, and parents. On the other hand, the teachers perceived that the compliments partly became superficial and reflected physical attributes when used with the children rather than personal characteristics, which was the intention of the activity. Teachers discussed how the challenges in adhering to the compliment activities were based on cultural differences between the Swedish and North American contexts. The teachers also identified that it was a challenge for themselves as individuals to convey a genuine use of compliments, as intended in PATHS, to the children. This led them to avoid certain complementary activities. The teachers perceived the intended use of compliments as somewhat foreign and discussed that it was important that they felt comfortable expressing compliments to convey this idea to the children.

“T4: one thing that has been difficult for me is that I, I mean I hear what [the trainers] say, I understand every word they say. But it still feels like there are quite big cultural differences, […] what [another teacher] said was that she cannot manage this giving compliments thing. […] Well, I grew up in [northern part of Sweden where people are said to be more reserved], and I don’t walk around [giving compliments], I mean, it feels like a British or American tradition (several laughs). […] this whole thing with compliments [is] lovely. But I haven’t done that. (Laughs). It is hard to adopt such a way of expressing yourself.

T2: It is funny that you say we have talked a lot about that. It has been kind of tough to get into this thing with compliments and how to do it, so that it feels natural. […] It feels very strange. When you said that, it was like, ‘Well, maybe that is why we felt that way’. Because it has been really tardy […]

T3: We sat there in the morning, where you should give yourself compliments and talk about what my strength is, and we are so bad at that. Like, ‘am I good at something? I am probably mediocre’”—FG 2.

### 3.3 Prerequisites within the new cultural context

The theme encompassed the teachers’ prerequisites in the form of both practical resources and collaboration with parents, which needed to be taken into greater consideration in the cultural adaptation of PATHS to the Swedish context.

#### 3.3.1 Resources within the Swedish preschool

The teachers described a vital and strained relationship with time in relation to implementing the culturally adapted PATHS in Swedish preschools. The relationship to time was discussed on the basis that Swedish preschool teachers’ working hours are filled with structured activities together with the children, where the preschool teachers have limited preparation time compared to, e.g., schoolteachers. In addition, any preparation time can also easily disappear due to circumstances such as staff absence. The preschool teachers perceived it as a constant negotiation about time and that it was their responsibility to create preparation time for each activity. Here, the teachers described different levels of support and understanding from the management at each preschool for the need of time. The responsibility for making time was perceived as problematic. It resulted in either using their own spare time in the evenings or weekends or asking for assistance from colleagues, which could generate a feeling of guilt that colleagues then had to do more of other work. Teachers also tried to find time to prepare PATHS activities during low-active work with the children present, such as during the younger children’s afternoon nap.

“T1: We do not have time, […] even though the management thinks we have many hours per week. […] But then it is usually like that, there are not enough people if we are to help out in different areas of the preschool […], so there is no allocated time [for preparation]. Instead, we have to make time for ourselves that does not exist in reality.

T2: And sometimes you feel guilty if you always have to ask [colleagues] to go in and do something. […so] I make time in the evening or sometimes during break time or something like that, I try. We sit outside with the kids when they are sleeping, so sometimes you try to sit there [and prepare PATHS] when you’re still working, but maybe not so active [with the children]”—FG 4.

The teachers also experienced that many projects come and go in Swedish preschools. The constant influx of new initiatives in Swedish preschools makes it difficult for interventions or work procedures to be maintained; there is always something new that requires energy and focus.

“T4: That [in preschool] you really try to improve things as much you can, but it can also be easy to feel forced or that you have to invent new things or keep up with new things. So, I guess there is a bit of a warning sign in that as well, that you still have to engage with things for a more extended time. Otherwise, it never has time to work fully. So, you have to hang in there to get these results, I think”—FG 1.

#### 3.3.2 Collaboration between teachers and parents in the Swedish preschool

The teachers pointed out that collaboration between teachers and parents is an important part of the children’s presence at Swedish preschools. This collaboration can pose a challenge which must be taken into regard when culturally adapting an intervention such as PATHS to the Swedish preschool context. Teachers felt unsure of how much parents carried out the parts of PATHS that they were intended to, such as complementary activities for the child in the home environment. In addition, teachers discussed the cultural expressions of parents and how parents introduced a perspective of emotions to their children, which at times could be contrary to what PATHS seeks to provide, e.g., different behaviors that are accepted for boys and girls with a father who never shows fear or becomes sad. At the same time, the teachers identified that it was essential that the work with PATHS came from both parents and teachers.

“T4: I think what you were talking about, that it is so different, that it works in one way at home, and another way here. [telling the child:]‘But I know you can put your boots on by yourself. Come on’, but as [another teacher] said, then maybe we still have to give the kids these options, that ‘Yeah, okay. At home, it works like that but here, and out in society, it works like this. Then you have to behave like this, and you have to treat other people this way’”—FG 1.

## 4 Discussion

This study explored aspects of cultural adaptation that needed further attention as perceived by teachers working with a culturally adapted preschool edition of PATHS in Sweden. The findings showed several aspects where cultural fit could be improved and possible further adaptations across the three identified themes pertained, overall, to the deliverers as persons and professionals with core values and understanding, and the deliverers’ perceptions of how the intervention aligned with fundamental values regarding children and preschool pedagogy, and prerequisites in the Swedish preschool context.

### 4.1 The need to consider the deliverer in cultural adaptation

Deliverers play a key role in making interventions available to those intended to benefit from it (i.e., end users and in the case of PATHS it would be preschool-aged children), which has been emphasized, and largely investigated, in the general field of implementation science (Chaudoir et al., 2013). Deliverers’ perceptions and attitudes toward an intervention may influence the extent to which the deliverers use the intervention (Aarons et al., 2012), thus whether the intervention is made available to end users. Another aspect of the deliverers’ use of the intervention comprises fidelity in intervention delivery. Low fidelity to delivery on the part of the deliverer is problematic as it compromises effectiveness on outcomes (Durlak and DuPre, 2008; Goble et al., 2021). Thus, it seems important to take the deliverers’ perspective into account in the process of culturally adapting an intervention to a new context to have the deliverer advocate and participate in intervention delivery with high fidelity. The attention given to the deliverers’ perspective within the field of cultural adaptation varies between different frameworks and theories. As examples of commonly used frameworks the ones by Bernal et al. (1995) and Castro et al. (2004) acknowledge the importance of aspects related to the deliverer, such as competence, but focus largely on adapting interventions to the cultural aspects of end users. Thus, the end user is the target and recipient of the cultural adaptation. One of the most recent and comprehensive guidelines for adaptation of interventions into new contexts, the ADPAT guidance, was developed to establish evidence and consensus-informed guidance for the adaptation process (Moore et al., 2021). The ADAPT guidance, like the PIA protocol (Ferrer-Wreder et al., 2012), provides systematic guidance to make adaptations in ways that facilitate an *a priori* and transparent adaptation processes. Both the ADPAT and the PIA guidelines highlight the need to include the practitioner/deliverer in the process of adapting an intervention. However, neither the PIA nor the ADAPT guidance puts the deliverer as the core target of the adaptation process. To advance the field of cultural adaptation of interventions, we suggest that frameworks that guide cultural adaptation integrate a perspective that focuses specifically on the deliverer as a core target/recipient of cultural adaptation as an equal part to the focus on end users. Here, established frameworks from the overall field of implementation science can be useful, as the perspective of the deliverer has been highlighted within this field (Chaudoir et al., 2013). An example of such a framework is the Consolidated Framework for Implementation Research (CFIR), which uses a multi-layered lens through which the implementation of interventions can be analyzed. The CFIR puts the deliverer of the intervention as the center of the multi-layered systems approach, surrounded by the organization, and societal factors when carrying out the specific intervention in focus (Damschroder et al., 2009). Furthermore, in recent years, the role of teachers in the implementation of SEL interventions has become more nuanced, than in the past, with wider recognition and now evidence to support the vital role that adults in schools play in conferring the intended benefits of SEL interventions to children and adolescents (e.g., Mihić et al., 2020; Shi and Cheung, 2024), with the need for whole school SEL interventions that support adults at school including teachers practically (e.g., FG participants comments are the need to support their preparation time) from an implementation standpoint as well as for teachers to have professional development space to grow in their own SEL (Shi and Cheung, 2024), which reflects the growing recognition of the SEL as having lifespan importance and value.

### 4.2 The need to consider core values in cultural adaptation and extend the adaptation process

Previous frameworks within the field of cultural adaptation have highlighted the need to make adaptations of interventions to align intervention with the core values of the new cultural context (Bernal et al., 1995; Castro et al., 2004). However, as much as adaptation is crucial, it may also be a time-and resource-intensive process.

The findings of this study that pertain to the use of compliments in the intervention provided an example where such effort was undertaken in the original cultural adaptation of preschool PATHS to the Swedish context (based on comments on the initial translation and adaptation of the sample lessons from the curriculum with a different FG of expert teachers), but is still in need of further adaptation. Although the cultural difference in the attitude toward and use of compliments between the Swedish and North American context had been identified in the original cultural adaptation of PATHS in Sweden (Ferrer-Wreder et al., 2021), this study showed that some teachers still found it difficult to implement compliment activities as described in the intervention, as some of the teachers perceived the compliment activity to be fundamentally different from their core values. This finding may, on the one hand, indicate that, in cases of intervention activities that stem from different fundamental core values, deliverers may need further training in how to perform the activities or need to be involved in how the activities should be adapted so that they perceive the practice as feasible. On the other hand, as core values and norms may to some extent be reflected on a surface level (e.g., visible compliments) but are often rooted in behaviors and values that are more or less taken for granted by individuals or groups, these may be more difficult to discern in a process that aims to identify aspects in need of cultural adaptation.

Hence, in addition to further training and involvement of the deliverers, the cultural adaptation process of preschool PATHS to a Swedish context may benefit from more than one round of cultural adaptation, although the culturally adapted intervention was adequately implemented and largely associated with the expected benefits (e.g., Eninger et al., 2021). The findings of this study indicate that the teachers in this study did not perceive the process of culturally adapting the PATHS intervention as complete, as they indicated several aspects in need of further adaptation. Just as several effectiveness evaluations are recommended to establish an intervention’s external validity in several contexts (Fraser, 2009), we argue that more than one round of cultural adaptation may be needed to establish a good fit between the intervention and the cultural context, particularly in the cross-national importation of interventions. A further argument for more than one round of cultural adaptation is the notion that cultural values, norms, and practices are not static in time but evolve in a dynamic process as a societal context, community, or group faces various events, situations, and conflicts. This would thus imply that cultural adaptations can be considered a circular process that evolves in parallel with cultural and societal development.

Finally, in their descriptions of the PATHS material and activities, the teachers sometimes described adaptations of a spontaneous nature. Such adaptations, or intervention drift, are suggested to compromise intervention effectiveness, whereas adaptations that are made *a priori* in a planned manner have instead been hypothesized to lead to increased or sustained effects of interventions (Chambers and Norton, 2016; Albers et al., 2020). Thus, several rounds of cultural adaptation of intervention could produce more detailed and planned adaptations described in detail for deliverers and thus facilitate intervention delivery without compromising fidelity.

### 4.3 Adapting interventions in cultural contexts that can be considered similar

This study considered a sample of teachers’ perceptions of a culturally adapted edition of preschool PATHS, which was developed in the United States and then adapted to be used in an urban Swedish preschool context. These are cultural contexts in two high-income Western countries. This study shows that a thorough cultural adaptation process is important even in contexts that, on a general level, can be perceived as having many similarities. Some of the teachers’ perceptions presented in the results section could be regarded as reflecting the underlying differences between Sweden and the US in terms of how ECEC has been developed and what educational values it is based on. In Sweden, as in other Nordic countries, ECEC is a crucial part of the Nordic welfare model and is based on democratic values such as solidarity, civil rights, equity, and equality (Broström et al., 2018), which is also reflected in the educational values of ECEC. These are also, both in the education of preschool teachers as well as ECEC itself, clearly influenced by the German perception of *Didaktik* as related to *Bildung* (Broström, 2022), rather than the Anglo-Saxon instruction-oriented didactics from which PATHS originates. This is a good example of the fact that in countries, that on a general level, share some similarities in financial and educational conditions, adaptation needs to be based on a more profound analysis of underlying societal prerequisites and values that have formed the practice for which the intervention is to be adopted.

## 5 Study limitations, strengths, and conclusion

While his study contributes to the field of cultural adaptation and implementation science in several ways, a number of study limitations should be noted. For example, this sample was not designed to be representative of all teachers who took part in the Swedish PATHS trial. The transferability of the findings from this study is limited to the sample included in the FGs and to the specific urban Swedish context described. Furthermore, the interview guide used in the data collection process was not specifically developed to explore cultural adaptation, and therefore aspects of relevance may have gone uncaptured in the FG discussions.

A strength of the study is that it is one of the first to explore how deliverers perceive a culturally adapted intervention. Thereby, the findings of the study may be beneficial to cultural adaptations of interventions in several contexts, not limited to Swedish or US settings. Further strengths of the study comprise the analysis process, where two researchers with extensive experience in qualitative methods analyzed the data to ensure trustworthiness. Furthermore, reporting quotes in the audit trail strengthens the confirmability of the findings. This study is also valuable in that it highlights the voices of key adults who have a definitive role to play in furthering SEL in their schools and in the context of their professional training and expertise.

### 5.1 Conclusion

From a wider theoretical and implementation perspective, the findings of this study highlight the importance of targeting the deliverer in the cultural adaptation process in addition to the end users to make the intervention culturally acceptable to the deliverer and thereby deliver the intervention in a way that confers intended benefits to end users. Further, findings point toward a need to extend the adaptation process to more than one round, when warranted by empirical findings from the cultural adaptation process, and possibly make it into a circular process that can disentangle underlying core cultural values and take the dynamics of evolving societies into account. In addition, the study highlights the importance of thoroughly considering the need for cultural adaptations in implementation settings, which at first sight may be considered to have many similarities on a surface level.

## Data availability statement

The datasets presented in this article are not readily available because qualified researchers (e.g., Ph.D.) who have ethical permission under Swedish law for secondary data analysis of this dataset can apply to access de-identified data in writing. Written requests should be directed to the PATHS/Parent Web Data Management Committee at PATHS_PW.Project@psychology.su.se.

## Ethics statement

The studies involving humans were approved by Stockholm Regional Ethics Review Board. The studies were conducted in accordance with the local legislation and institutional requirements. The participants provided their written informed consent to participate in this study.

## Author contributions

ÅN: Writing – review & editing, Writing – original draft, Methodology, Formal analysis, Data curation, Conceptualization. MS: Writing – review & editing, Writing – original draft, Methodology, Formal analysis, Data curation, Conceptualization. LF-W: Writing – review & editing, Writing – original draft, Validation, Funding acquisition, Conceptualization. LE: Writing – review & editing, Writing – original draft, Validation, Funding acquisition, Conceptualization. HH: Writing – review & editing, Writing – original draft, Validation, Conceptualization.
