# Supplementary material for: Insights gained from a cultural adaptation of preschool promoting alternative thinking strategies©: the importance of teachers’ cultures as an implementation driver
Source: Front Psychol. 2024 Aug 7;15:1425936. doi: 10.3389/fpsyg.2024.1425936 (PMC11336572; doi:10.3389/fpsyg.2024.1425936)
Supplement: Supplementary file 1 [file Data_Sheet_1.pdf]

Supplementary material 1

Interview guide for focus groups

- How did you perceive the work with PATHS overall?
- How have you adapted PATHS?
- What were the benefits and challenges when working with PATHS?
- What helped you to do PATHS?
- What has been a barrier to doing PATHS?
- What do you think about the support you have gotten to do PATHS?
- What possibility did you have to integrate the basic ideas of PATHS with you daily work routine with the children?
- What do you think about taking on new ways of working?
- What resources are needed to implement PATHS in preschools in a manner that would make substantial difference for the children?
- Do you think you will keep a focus on social emotional learning in the future?
